# *Enterococcus* Species in the Oral Cavity: Prevalence, Virulence Factors and Antimicrobial Susceptibility

**DOI:** 10.1371/journal.pone.0163001

**Published:** 2016-09-15

**Authors:** Edson Yukio Komiyama, Laura Soares Souto Lepesqueur, Cinthia Gomes Yassuda, Lakshman P. Samaranayake, Nipuna B. Parahitiyawa, Ivan Balducci, Cristiane Yumi Koga-Ito

**Affiliations:** 1 Department of Oral Biosciences and Diagnosis, Oral Biopathology Graduate Program, Institute of Science and Technology, Universidade Estadual Paulista/UNESP, São José dos Campos, São Paulo, Brazil; 2 UQ Oral Health Centre, School of Dentistry, The University of Queensland, Brisbane, Queensland, Australia; 3 Department of Oral Bio-Sciences, Faculty of Dentistry, The University of Hong Kong, Hong Kong, SAR, China; 4 Department of Social Dentistry, Institute of Science and Technology, Universidade Estadual Paulista/UNESP, São José dos Campos, São Paulo, Brazil; 5 Department of Environmental Engineering, Institute of Science and Technology, Universidade Estadual Paulista/UNESP, São José dos Campos, São Paulo, Brazil; Universidad Nacional de la Plata, ARGENTINA

## Abstract

Enterococci are considered as transient constituent components of the oral microbiome that may cause a variety of oral and systemic infections. As there is sparse data on the oral enterococcal prevalence, we evaluated the *Enterococcus* spp. and their virulence attributes including antimicrobial resistance in a healthy Brazilian cohort. A total of 240 individuals in different age groups were studied (children 4–11 yrs, adolescents 12–17 yrs, young adults 18–29 yrs, adults 30–59 yrs, elderly over 60 yrs). Oral rinses were collected and isolates were identified by API 20 Strep and confirmed by 16S rDNA sequencing. *E*. *faecalis* isolates, in particular, were evaluated for virulence attributes such as their biofilm formation potential, and susceptibility to antimicrobials and an antiseptic, chlorhexidine gluconate. A total of 40 individuals (16.6%) and 10% children, 4% adolescents, 14% young adults, 30% adults, and 25% elderly carried oral enterococci. The oral enterococcal burden in adolescents was significantly lower than in the adults (p = 0.000) and elderly (p = 0.004). The proportion of carriers was higher among females (p = 0.001). *E*. *faecalis* was the most frequent isolate in all the age groups (p = 0.000), followed by *E*. *durans* and *E*. *faecium*. Whilst all the clinical isolates were able to form biofilms, only a proportion of them were able to produce lipase (92%), hemolysin (38%), and gelatinase (39%). Of all the isolates 53.8% were resistant to tetracycline, 12.3% to amoxicillin, 16.0% to ampicillin, 20.8% to chloramphenicol and 43.4% to erythromycin. None of the isolates were resistant to vancomycin. Our data suggest that in this Brazilian cohort the oral cavity may act as a significant reservoir of rather virulent and antibiotic resistant enterococci, with an increasing degree of carriage in the adults and elderly. Hence clinicians should be cognizant of this silent reservoir of virulent enterococci that may pose a particular threat of nosocomial infection.

## Introduction

It is well known that enterococci cause several infections some of which are potentially fatal, including urinary tract, neonatal and wound infections, as well as endocarditis and meningitis [1,2,3,4]. Over the last few decades, they have also been recognized as notable nosocomial pathogens worldwide, in particular due to the increasing emergence of antimicrobial resistance phenotypes [5,6,7,8]. Noteworthy are the vancomycin-resistant enterococci, and their impact in nosocomial settings that are of considerable concern [2,4,5,9]. Apart from vancomycin, enterococci also exhibit resistance to tetracycline, penicillin, cephalosporin and aminoglycosides [10,11].

*E*. *faecalis*, the predominant human enterococcus, has been also related to oral diseases, such as caries, endodontic infections, periodontitis, and peri-implantitis [12,13,14]. It has been frequently implicated in failure of endodontic treatment, due to high resistance to endodontic medicaments, and the ability to form recalcitrant biofilms both in treated and untreated root canals [15,16,17,18,19].

Although enterococci are generally considered as transient constituents of the oral microbiome with a low colonization density [20,21], surprisingly little is known about their oral prevalence and incidence particularly in different population groups [22]. Most of the currently available data pertain to the oral carriage of *E*. *faecalis* mainly in patients undergoing endodontic therapy, and it is considered as one of 25 most abundant pathogens causing persistent endodontic infections [18,23]. On the contrary, patients with gingivitis or periodontitis exhibit an enterococcal prevalence ranging from 3.7 to 35% [12,24]. Interestingly, Chomicz et al. [25] reported that 60% of diabetic patients yielded oral *Enterococcus fecalis*, *and E*. *faecium*, as opposed to only 6.6% in the controls.

Recently, Anderson et al. [23] in an elegant study comprehensively evaluated the virulence attributes of *E*. *faecalis* isolates from the oral cavity, food and clinical specimens, and reported that oral isolates had the highest percentages of virulence genes as well as extracellular enzymes and a capacity to form biofilms. They suggested, therefore, that the oral cavity may constitute a critical reservoir of virulent, antibiotic resistance *E*. *faecalis* strains.

Hence, the primary aim of this study was to determine the oral prevalence of *Enterococcus* spp. in different age groups, in a healthy Brazilian cohort. A secondary aim was to evaluate the major virulence traits of the *E*. *faecalis* oral isolates (biofilm formation and enzyme production) and their susceptibility to vancomycin, amoxicillin, tetracycline, ampicillin, chloramphenicol, erythromycin, and a commonly used antiseptic chlorhexidine digluconate.

## Materials and Methods

### Ethics Statement

The study was approved by the Universidade Estadual Paulista Institute of Science and Technology, Local Institutional Ethics Committee (protocol number 060/06-PH/CEP). The volunteers were fully informed about the aims of the study and methods of sampling. All adult subjects provided written informed consent, by signing a form. In case of children, parent or guardian provided written informed consent on their behalf, respecting child’s decision to participate. Additionally, volunteers who donate blood used for haemolysin activity detection also gave written informed consent by signing a form.

A total of 240 healthy volunteers, 88 males (36.67%) and 152 females (63.33%), attending the clinics at São José Institute of Science and Technology, Brazil, were sampled. The cohort was divided into five age groups as follows: i) children: 4–11 years-old (n = 50); ii) adolescents: 12–17 years-old (n = 50), young adults: 18–29 years-old (n = 50), adults: 30–59 years-old (n = 50), elderly: over 60 years-old (n = 40). The individuals were randomly selected using a table of serial numbers of patient records.

Patients with diabetes mellitus, pregnant women, smokers, and orthodontic appliances/denture users were excluded from the study as well as those on treatment with antimicrobials/antifungals and/or mouthwashes or other medications that affect the salivary flow over last 60 days prior to the study.

Each individual was sampled using the oral rinse technique using 10 mL of phosphate buffered saline (PBS, 0.1M, pH 7.2) [26]. The patient was asked to rinse the mouth with PBS, for 1 min, and expectorate the rinse into a sterile container. In the laboratory, the samples were centrifuged for 10 min at 8,000 X g and the supernatant was discarded, and 2.5 ml of PBS was added to the cell pellet that was reconstituted. An aliquot (0.1 ml) of each suspension was plated on Enterococcosel Agar (BBL, France) and aerobically incubated at 37°C for 24 h. Afterwards, the characteristic colonial growth (with brown halos) was quantified as the number of colony-forming units per milliliter (cfu/mL) of oral rinse. Five representative colonies of different morphologies were further evaluated by microscopy, and transferred into Tryptic Soy broth (TSB—Difco, Detroit, USA) and incubated at 37°C for 24 h. Then, an aliquot of 0.1 ml from the resultant growth was stored in 2 ml of Brain Heart Infusion broth-glycerol (BHI-glycerol) (20%) and maintained at -80°C prior to final identification.

All the isolates were phenotyped using the API 20 Strep system, according to the manufacturer’s instructions (Biomérieux, Marcy l’Etoile, France). Subsequently, 16S rDNA sequencing was performed for molecular confirmation of the identity. Briefly, cultures were grown in Triptic Soy agar overnight at 37°C and a colony was suspended in 50 μL of sterile miliQ water and heated at 95°C for 10 min in a thermal cycler (Applied Biosystems, ABI 2100). An aliquot of 2 μL of the mixture was used for PCR.

For PCR, following primer pair was used: D88 (5’-GAGAGTTTGATYMTGGCTCAG-3’) and E 94 (5’-GAAGGAGGTGWTCCARCCGCA-3’). The PCR amplification was carried out in 50 μl final volume containing 10 μM of each primer, 4 μl of PCR buffer, 1.2 μl of 50mM MgCl_2_, 0.6 μl of 10mM dNTP mix, 0.14 μl of 5U/μl *Taq* DNA polymerase, 30.86 μl ultra-pure water and 2 μl of DNA template. Cycling conditions consisted of 3 min at 95°C followed by 35 cycles of 45 s at 94°C, 1 min at 53°C, 90 s at 72°C, followed by 72°C for 7 min. PCR products were evaluated in 1% agarose gel and positive products purified using QIAGEN PCR purification kit. Sequencing reaction was performed using ABI 3730 sequencer (Applied Biosystems). The primers D88 and E94 were used as sequencing primers in BigDye version 3.1 reaction. The chromograms were visually inspected for quality and length of reads. Poor quality results were discarded. The sequences were compared against all non-redundant bacterial sequences deposited in GenBank using BLAST.

Isolates identified as *E*. *faecalis* (n = 100) were evaluated for virulence traits (enzyme production) and susceptibility to antimicrobials. For this, the frozen, aliquoted isolates were thawed and inoculated on BHI agar plates, and incubated at 37°C for 24 hours. After confirmation of purity, the following tests were performed in duplicate in two different occasions.

Haemolysin activity was evaluated by inoculation in Müeller-Hinton agar supplemented with 5% of human total blood (v/v) (see Ethics Statement Section), as previously reported [27,28]. The presence of a clear halo (β-hemolysis) around the colonies after incubation for 24 hours at 37°C was considered as positive for haemolysin.

Gelatinase production was evaluated using Triptic soy agar supplemented with 3% gelatin, as described before [27,29]. After inoculation, the plates were incubated at 37°C for 48 hours and the presence of clear halo around the colonies was considered as gelatinase positive.

Lipase production was assessed according to the methodology proposed by Elsner et al. [30]. In brief, isolates were plated on the surface of Spirit Blue agar supplemented with 3% Bacto lypase reagent and incubated at 37°C for 72 hours. An opaque zone around the colonies indicated lypolytic activity.

Lipase, haemolysin and gelatinase production was scored qualitatively as either positive or negative and quantification was not attempted.

Biofilm formation and evaluation of susceptibility to chlorhexidine digluconate were performed according to previously described methodology [31,32]. Standardized bacterial suspensions (1x 10^8^ cells/ml) in physiologic saline (NaCl 0.9%) of each *E*. *faecalis* isolate (n = 100) were prepared. Then, an aliquot of 20 μl of the bacterial suspension was transferred to cellulose membranes and placed on the surface of BHI agar. Plates were aerobically incubated for 48 hours at 37°C, and evaluated for biofilm formation. To evaluate the effect of chlorhexidine digluconate, the membranes were transferred to a sterile Petri dish containing 5 ml of 0.12% chlorhexidine digluconate. After 1 minute of exposure to the antisepic, the membranes were first transferred to plates containing 5 ml of a neutralizer for 1 minute (0.5% Tween 80 supplemented with 0.07% lecithin) and then into a sterile physiologic solution and vortexed for 60 seconds to disrupt the biofilm. Serial ten-fold dilutions of the latter suspension were obtained and 0.1 ml was plated in duplicate in BHI agar. Plates were incubated under aerobic conditions at 37°C for 48 hours and the number of colony-forming units (cfu) per membrane was calculated. Negative control samples were immersed in physiologic solution under identical conditions.

Minimal inhibitory concentrations (MIC) of amoxicillin, ampicillin, tetracycline, vancomicin, chloramphenicol, and erythromycin were determined by agar dilution method according to Clinical Laboratory Standards Institute (CLSI) guidelines [33]. The breakpoints for resistance adopted were those established by the CLSI guidelines (chloramphenicol ≥ 32μg/ml, erythromycin ≥ 8 μg/ml, amoxicillin ≥ 16μg/ml, ampicillin ≥ 16μg/ml, tetracycline ≥ 16 μg/ml, vancomycin ≥ 32 μg/ml).

Data were statistically analyzed by GraphPad PRISM version 6. Differential counts of enterococci between the various age groups were, expressed as cfu per milliliter, and compared by Kruskal Wallis and Dunn’s test. The prevalence of enterococci between males and females was analyzed using Z test. The bactericidal effect of chlorhexidine digluconate on *E*. *faecalis* biofilms were analyzed using Mann-Whitney test. p < 0.05 was considered statistically significant for all the evaluations.

## Results

A total of 40 individuals (16.6%) in our cohort were carriers of oral enterococci, with a mean count of 122 cfu/mL amongst the carriers. Specifically, 10 per cent of children (mean = 88 cfu/ml), 4% for adolescents (1 cfu/ml), 14% for young adults (3.5 cfu/ml), 30% of adults (216 cfu/ml), and 25% of elderly (224 cfu/ml) were positive for enterococci. Significant difference in enterococcal carriage was observed between the adolescents, and the adults (p = 0.000), and elderly (p = 0.004) (Table 1). Higher proportion of carriers was observed among females (63.33%) than males (36.67%) (p = 0.001).

**Table 1:** Oral carriage of enterococci in different age-related cohorts (expressed as colony-forming units per milliliter; cfu/mL).

Groups	n	% of positive^§^	mean	sd	median	min	max
Children	50	10	88	344.70	0	0	2050
Adolescents*^,^^♣^	50	4	1	4.95	0	0	25
Young adults	50	14	3.50	8.76	0	0	25
Adults*	50	30	216	954	0	0	6425
Elderly^♣^	40	25	224	670	0	0	2875

n = number of patients, sd = standard deviation, min = minimum value, max = maximum value.

*p = 0.000,

^♣^p = 0.004;

^§^ percentage of individuals positive to *Enterococcus* spp. in the oral cavity

The species distribution of enterococci in each group is shown in Table 2. Of the 115 enterococcal isolates, overall the most predominant were *E*. *faecalis* (88.7 per cent) followed by *E*. *durans* (7.9%) and *E*. *faecium* (1.7%). *E*. *faecalis* carriage for children, adults and elderly were 94.1%, 89.5%, 81.6%, respectively. *E*. *durans* was the second most frequently isolated species representing 7.9% of the isolates. The latter was most frequently found in elderly patients (15.8%), followed by adults (4.2%) and children (5.96%). *E*. *durans* was not isolated from either adolescents or young adults. A very low prevalence of *E*. *faecium* was noted in adults with only two isolation events (1.7%).

**Table 2:** Distribution of *Enterococcus* species in each age group.

Age group	*E*. *faecalis*	*E*. *faecium*	*E*. *durans*	*Enterococcus* sp.(unassigned)	Total
Children	16	0	1	0	17
Adolescents	2	0	0	0	2
Young adults	10	0	0	0	10
Adults	43	2	2	1	48
Elderly	31	0	6	1	38
Total	102 (88.7%)	2(1.7%)	9(7.9%)	2(1.7%)	115

We also noted marked differences when the identical isolate was phenotyped by the API 20 Strep system and genotyped by rDNA sequence analyses, with 21.7% (25 of the total) of isolates being assigned to two different species (Table 3). For instance, the genotype *E*. *faecalis* was identified by API 20 Strep system as *E*. *avium*, *E*. *durans*, *E*. *faecium* in 14 analyzed samples, whilst the genotype *E*. *durans* was identified as *E*. *faecalis* and *E*. *faecium* by API 20 Strep on two occasions. We therefore used the rDNA sequence analyses results as definitive information for the current study.

**Table 3:** Phenotypic (API 20 Strep method) and genotypic (16s rDNA) analytical data of 25 oral enterococcal isolates indicating the divergent nature of the results.

Sequencing	n	Homology*	API 20 Strep
*E*. *faecalis*	1	98%	*E*. *durans*
*E*. *durans*	7	99–100%	*E*. *faecalis*
*E*. *avium*	4	100%	*E*. *faecalis*
*E*. *faecium*	3	99–100%	*E*. *faecalis*
*E*. *avium*	2	100%	*E*. *faecalis*
*E*. *avium*	1	99%	*Enterococcus* sp.
*E*. *faecium*	6	99–100%	*E*. *durans*
*E*. *faecium*	1	99%	*Enterococcus* sp.

*Percentage sequencing identity with reference sequences in GenBank.

In terms of the virulence attributes, all *E*. *faecalis* isolates were able to form biofilms, and variable percentages of isolates were able to produce lipase (92%), hemolysin (38%), and gelatinase (39%). Chlorhexidine digluconate had significantly reduced the number of viable cells in all *E*. *faecalis* biofilms evaluated (p = 0.008<0.05).

Breakpoint antimicrobial resistance profiles of all the isolates are shown in Table 4. Of the total of isolates (n = 115), 53.8% were resistant to tetracycline, 43.4% to erythromycin, 20.8% to chloramphenicol, 16.0% to ampicillin, and 12.3% to amoxicillin. None of the isolates were resistant to vancomycin.

**Table 4:** Break point antimicrobial sensitivity profiles (values in μg/ml) of oral *E*. *faecalis*, *E*. *faecium* and *E*. *durans* spp.

Antimicrobial	*Enterococcus* spp. (n = 115)	*Enterococcus* species		
		*E*. *faecalis* (n = 104)	*E*. *faecium*(n = 2)	*E*. *durans*(n = 9)
**Amoxicillin**				
MIC90	32	4	> 64	8
MIC50	2	2	2	2
Range	1–64	1–64	1–64	1–8
% Resistance	12.3	16	6	100
**Ampicillin**				
MIC90	16	4	16	8
MIC50	4	8	8	2
Range	1–64	1–64	4–16	2–8
% Resistance	16.0	18	37	100
**Tetracycline**				
MIC90	64	>64	> 64	64
MIC50	16	16	4	4
Range	1–64	1–64	1–64	2–64
% Resistance	53.8	54	38	50
**Vancomycin**				
MIC90	8	4	1	4
MIC50	1	1	1	1
Range	1–8	1–4	1	1–8
% Resistance	0	0	0	0
**Chloramphenicol**				
MIC90	32	32	32	64
MIC50	16	16	16	16
Range	1–64	1–64	2–64	2–64
% Resistance	20.8	20	10	33
**Erythromycin**				
MIC90	64	> 64	> 64	> 64
MIC50	4	4	8	1
Range	1–64	1–64	1–64	1–64
% Resistance	43.4	46	56	17

## Discussion

Enterococci are generally considered as transient oral bacteria [21]. However, their prevalence in various population groups has not been fully evaluated, with only sparse data mainly from North American and European studies [20,34]. To our knowledge, this is the first study to report the prevalence of oral enterococi from a South American cohort belonging to a spectrum of age groups. As we intended to study a sample representative of the general population the exclusion criteria included only the most frequently reported conditions that affect the oral microbiome such as, diabetes, pregnancy, wearing orthodontic appliances and dentures, use of mouth rinses, and medications that affected the salivary flow.

In our cohort, 10% of children, 4% of adolescents, 14% of young adults, 30% of adults and 25% of elderly were oral carriers of enterococci. The overall 16.6% oral prevalence of enterococci reported here is similar to those with a healthy gingival and periodontal status (20%), but much lower than for patients with gingivitis/periodontitis (73%) and undergoing endodontic treatment in a dental institute in USA [34], as well as in a cohort of insulin-dependent diabetic patients from Europe (60%) [25]. The latter groups have also reported a much lower prevalence (1%) in dental students [35] and in healthy individuals (6.6%) [25]. It is noteworthy, however, that we did not evaluate periodontal status nor the endodontic diseases in our cohort. As almost all of the above studies including ours, report a relatively high intra oral prevalence of *E*. *faecalis*, further longitudinal studies on the incidence and oral carriage of these organisms are warranted to obtain a complete picture of the oral colonization profiles of enterococci.

Several previous workers have used molecular methods rather than phenotypic ones, yet limited their studies for investigating only *E*. *faecalis* species [35,36,37]. However, considering that other enterococci such as *E*. *faecium*, have emerged as important pathogens particularly in nosocomial environments [38], we aimed to identify all enterococci isolated. The picture that is emerging is worthy of note as it seems that several species of enterococci routinely habituate the oral cavity, the implications of which are yet to be defined.

As the sampling method we adopted was the oral rinse technique generally acknowledged as the most effective technique for microbiological screening of the healthy oral cavity [26] we surmise that our data present a relatively accurate picture of the oral enterococcal profile in our population. We also performed pilot experiments using several media for standardization of *Enterococcus* isolation using bile esculin agar (Himedia, India), Enterococcus agar (Difco, Detroit, USA) and Enterococcosel agar (BD, France). Oral rinses samples were simultaneously plated on the foregoing media and the resultant growth was evaluated after 18 hours of incubation. As enterococal agar yielded the most CFUs presumptive of enterococci, as characterized by a brown halo around each colony, we decided to use the latter culture medium for our subsequent experiments (data not shown).

It is known that the phenotypic speciation methods of streptococci such as ID32Strep, API 20 Strep and VITEK2 systems [39,40] lead to spurious results compared with genotypic speciation methods such as *mpB* analysis. For instance, Winston et al. [41] compared the results of *rnpB* analysis and API20 Strep system and demonstrated a high incidence of incorrect identification of enterococcal species by the latter technique. Hence, we used both the phenotypic and genotypic identification systems and noted only a 78.3 per cent concordance in the results with 25 strains identified discordantly as follows (Table 3). The genotype *E*. *faecalis* was identified as *E*. *avium*, *E*. *durans*, *E*. *faecium* by API 20 Strep in 14 analyzed samples, whilst *E*. *durans* was phenotyped as *E*. *faecalis* and *E*. *faecium* by API 20 Strep on two occasions. The significant variation in the two typing systems implies that the more accurate and reliable genotyping system, rather than the traditional phenotyping systems, should be used by future workers to yield consistent, reliable and comparable data in epidemiologic studies of this nature.

In quantitative terms of enterococcal colonization we noted a mean density of 122 cfu/mL amongst the carriers with particularly high values of up to 224 cfu/mL in the elderly. These values are considerably lower than those of Sedgley et al. [27] who reported 10 to 600 cfu/mL, and 1000 to 4600 cfu/mL in two different studies. This difference could be attributed to the demographic variations, the oral health and the methodology between these studies. For instance, Sedgley et al. [27] essentially evaluated a relatively small sample of eleven patients who were undergoing endodontic treatment and used bile esculin agar as opposed to Enterococal agar use in the current studies. Both groups, however, employed oral rinses as the standard sampling methodology. On the other hand, in a subsequent study, Sedgley et al. [35] reported enterococcal counts between 30 to 240 cfu/mL in patients undergoing endodontic treatment.

To the best of our knowledge this is the first report of gender variations in oral carriage of enterococci. We also noted significant, age-related differences of oral enterococcal prevalence in this study. The origin of the enterococci in the oral cavity is yet unclear. Some contend that most enterococci enter the oral cavity as contaminants derived from normal intestinal flora, whilst person to person translocation or transfer through water or contaminated food have been suggested by others [42,43]. Interestingly, a number of workers have surmised that antibiotic resistant enterococci in particular can be transmitted through contaminated food, leading to infections [43,44]. However, more recently, Vidana et al. [45] found no evidence of foodborne transmission of enterococci.

Of the enterococcal species, *E*. *faecalis* is the most frequently isolated species in previous studies including ours with *E*. *faecium* closely following. These two species comprised 80% of our clinical isolates. There are no previous reports on oral enterococcal carriage amongst individuals in different age groups either in health or disease, and more work is required to confirm or refute the current data.

One curious finding in our study was the relatively high percentage of antibiotic resistant isolates compared to previous studies. A significant percentage of the isolates were particularly resistant to tetracycline, chloramphenicol and erythromycin. This observation contrasts with that of Chai et al. [31] who after examining *E*. *faecalis* ATCC 29212 surmised that erythromycin and tetracycline were totally effective for eliminating *E*. *faecalis* from oral biofilms. Sedgley et al. [27] on the other hand analyzed 12 oral enterococci and detected only two tetracycline resistant isolates. Though rather tenuous, one reason for the origin of tetracycline resistance in oral enterococci could be its frequent administration in periodontitis patients [46]. Concurring with our data, Anderson et al. [23] have very recently reported relatively high frequency of erythromycin-resistant *E*. *faecalis* from food, clinical specimens and oral sites. Variations in antimicrobial profiles and emergence of antimicrobial-resistant bacteria in diverse geographic regions are relatively common due to the differing antibiotic prescribing practices in the respective locales, including overprescribing. As our report is the first to evaluate antimicrobial resistance profile of oral enterococci from South America, and in particular Brazil, our findings need to be further examined by evaluating a much larger number of oral streptococci. Until further data are available it is instructive to note the high degree (>50%) of tetracycline-resistant *E*. *faecalis* in our isolates, as they could pose a threat to spread of nosocomial infection particularly in patients in intensive care units, and those on mechanical ventilators. Our findings that chlorhexidine digluconate (0.2%), a common ingredient in various mouth washes, was effective in suppressing *E*. *faecalis* may have clinical implications here as this antiseptic is widely used for oral hygiene for patients in long term ambulatory care.

Finally, we also noted that all the enterococcal isolates in our study were able to develop biofilms, and most had the capacity to produce virulence-related enzymes. As the current data are qualitative in nature much more work on the virulence attributes of oral enterococci needs to be performed to characterize oral enterococci and their pathogenic potential including whole genome sequence analysis. Nevertheless, our data underscore and further confirm that the commensal oral enterococcal populations have the requisite virulence attributes including resistance to commonly prescribed antibiotics, to make them ideal candidate organisms that could cause either oral or systemic disease. Whilst our data highlight the need for appropriate oral care in nosocomial settings, further studies with larger numbers of isolates form various geographic settings are essential to obtain a complete picture of the oral enterococcal distribution in various population groups and their virulence attributes.

## References

[1] Colodner R, Eliasberg T, Chazan B, Raz R. Clinical significance of bacteriuria with low colony counts of Enterococcus species. Eur J Clin Microbiol Infect Dis. 2006;25: 238–41. 16596356 10.1007/s10096-006-0132-0

[2] Butler KM. Enterococcal infection in Children. Semin Ped Infect Dis 2006;17: 128–139. 10.1053/j.spid.2006.06.006 16934707

[3] Flores-Mireles AL, Walker JN, Caparon M, Hultgren SJ. Urinary tract infections: epidemiology, mechanisms of infection and treatment options. Nat Rev Microbiol. 2015;13: 269–84. 10.1038/nrmicro3432 25853778 PMC4457377

[4] Chirouze C, Athan E, Alla F, Chu VH, Ralph Corey G, Selton-Suty C et al Enterococcal endocarditis in the beginning of the 21st century: analysis from the International Collaboration on Endocarditis-Prospective Cohort Study. Clin Microbiol Infect. 2013;19:1140–7. 10.1111/1469-0691.12166 23517406

[5] Lavigne JP, Marchandin H, Czarneeki E, Kaye C, Sotto A. Bactériémies à *Enterococcus* spp.: etude prospective au CHU de Nimes Enterococcal bacteremia at Nîmes university hospital. J Pathol Biol. 2005;53: 539–45. 10.1016/j.patbio.2005.06.005 16084034

[6] Gilmore MS, Lebreton F, van Schaik W. Genomic transition of enterococci from gut commensals to leading causes of multidrug-resistant hospital infection in the antibiotic era. Curr Opin Microbiol. 2013;16(1):10–6. 10.1016/j.mib.2013.01.006 23395351 PMC3649759

[7] Chowdhury SA, Nallapareddy SR, Arias CA, Murray BE. The majority of a collection of U.S. endocarditis *Enterococcus faecalis* isolates obtained from 1974 to 2004 lack capsular genes and belong to diverse, non-hospital-associated lineages. J Clin Microbiol. 2014;52: 549–56. 10.1128/JCM.02763-13 24478487 PMC3911312

[8] Mittal S, Singla P, Deep A, Bala K, Sikka R, Garg M et al Vancomycin and High Level Aminoglycoside Resistance in *Enterococcus* spp. in a Tertiary Health Care Centre: A Therapeutic Concern. J Pathog. 2016;2016:8262561 10.1155/2016/8262561 27047693 PMC4800106

[9] Leavis HL, Bonten MJM, Willems RJL. Identification of high-risk enterococcal clonal complexes: global dispersion and antibiotic resistance. Curr Op Microbiol. 2006;9: 1–7. 10.1016/j.mib.2006.07.001 16880002

[10] Kirschner C, Maquelin K, Pina P, Ngo Thi NA, Choo-Smith LP, Sockalingum GD et al Classification and identification of enterococci: a comparative phenotypic, genotypic, and vibrational spectroscopic study. J Clin Microbiol. 2001;39(5):1763–70. 11325987 10.1128/JCM.39.5.1763-1770.2001 PMC88022

[11] Silva JA, Loyola PS, Galleguillos JO, Rodriguez YG, Colque-Navarro P, Möllby R, Kuhn I. Prevalencia de enterococos resistentes a antibióticos em aguas servidas em el norte de Chile. Rev Med Chile. 2005;133: 1201–1210. 16341371 10.4067/s0034-98872005001000009

[12] Kouidhi B, Zmantar T, Mahdouani K, Hentati H, Bakhrouf A. Antibiotic resistance and adhesion properties of oral Enterococci associated to dental caries. BMC Microbiol. 2011;11: 155 10.1186/1471-2180-11-155 21714920 PMC3150259

[13] Dahlèn G. Role of suspected periodontopathogens in microbiological monitoring of periodontitis. Adv Dent Res. 1993;7: 163–74. 8260004 10.1177/08959374930070020701

[14] Rams TE, Feik D, Mortensen JE, Degener JE, van Winkelhoff AJ. Antibiotic susceptibility of periodontal *Enterococcus faecalis*. J Periodontol. 2013;84: 1026–33. 10.1902/jop.2012.120050 23106507

[15] Sedgley C, Molander A, Flannagan SE, Nagel AC, Appelbe OK, Clewell DB, Dahlén G. Virulence, phenotype and genotype characteristics of endodontic *Enterococcus* spp. Oral Microbiol Immunol. 2005;20: 10–9. 15612939 10.1111/j.1399-302X.2004.00180.x

[16] Duggan JM, Sedgley CM. Biofilm formation of oral and endodontic *Enterococcus faecalis*. J Endod. 2007;33: 815–818. 17804318 10.1016/j.joen.2007.02.016

[17] Appelbe OK, Sedgley CM. Effects of prolonged exposure to alkaline pH on *Enterococcus faecalis* survival and specific gene transcripts. Oral Microbiol Immunol. 2007;22: 169–174. 17488442 10.1111/j.1399-302X.2007.00340.x

[18] Al-Ahmad A, Müller N, Wiedmann-Al-Ahmad M, Sava I, Hübner J, Follo M et al Endodontic and salivary isolates of *Enterococcus faecalis* integrate into biofilm from human salivary bacteria cultivated in vitro. J Endod. 2009;35: 986–91. 10.1016/j.joen.2009.04.013 19567320

[19] Ran S, Wang J, Jiang W, Zhu C, Liang J. Assessment of dentinal tubule invasion capacity of *Enterococcus faecalis* under stress conditions ex vivo. Int Endod J. 2015;48: 362–72. 10.1111/iej.12322 24872016

[20] Razavi A, Gmür R, Imfeld T, Zehnder M. Recovery of *Enterococcus faecalis* from cheese in the oral cavity of healthy subjects. Oral Microbiol Immunol. 2007;22: 248–251. 17600536 10.1111/j.1399-302X.2006.00349.x

[21] Zehnder M, Guggenheim B. The mysterious appearance of enterococci in filled root canals. Int Endod J. 2009;42: 277–287. 10.1111/j.1365-2591.2008.01537.x 19220511

[22] Aas JA, Paster BJ, Stokes LN, Olsen I, Dewhirst FE. Defining the normal bacterial flora of the oral cavity. J Clin Microbiol. 2005;43: 5721–32. 16272510 10.1128/JCM.43.11.5721-5732.2005 PMC1287824

[23] Anderson AC, Jonas D, Huber I, Karygianni L, Wölber J, Hellwig E et al *Enterococcus faecalis* from Food, Clinical Specimens, and Oral Sites: Prevalence of Virulence Factors in Association with Biofilm Formation. Front Microbiol. 2016;6: 1534 10.3389/fmicb.2015.01534 26793174 PMC4707231

[24] Sun J, Sundsfjord A, Song X. *Enterococcus faecalis* from patients with chronic periodontitis: virulence and antimicrobial resistance traits and determinants. Eur J Clin Microbiol Infect Dis. 2012;31: 267–272. 10.1007/s10096-011-1305-z 21660501

[25] Chomicz L, Szubinska D, Piekarczyk J, Wojtowicz A, Piekarczyk B, Starosciak B et al Occurrence of subclinical infections of the oral cavity in the insuline treated diabetics. Wiad Parazytol. 2004;50: 177–180. 16859022

[26] Samaranayake LP, Mc Farlane TW, Lamey PJ, Ferguson MM. A comparison of oral rinse and imprint sampling techniques for the detection of yeasts, coliform and *Staphylococcus aureus* carriage in the oral cavity. J Oral Pathol. 1998;15:286–288. 10.1111/j.1600-0714.1986.tb00646.x 3098945

[27] Sedgley C, Lennan SL, Clewell DB. Prevalence, phenotype and genotype of oral enterococci. Oral Microbiol Immunol. 2004;19: 95–101. 14871348 10.1111/j.0902-0055.2004.00122.x

[28] Creti R, Imperi M, Bertuccini L, Fabretti F, Orefici G, Di Rosa R et al Survey for virulence determinants among *Enterococcus faecalis* isolated from different sources. J Med Microbiol. 2004;53: 13–20. 14663100 10.1099/jmm.0.05353-0

[29] Salah R, Dar-Odeh N, Abu Hammad O, Shehabi AA. Prevalence of putative virulence factors and antimicrobial susceptibility of Enterococcus *faecalis* isolates from patients with dental Diseases. BMC Oral Health. 2008; 8:17 10.1186/1472-6831-8-17 18513445 PMC2424041

[30] Elsner HA, Sobottka I, Mack D, Claussen M, Laufs R, Wirth R. Virulence factors of *Enterococcus faecalis* and *Enterococcus faecium* blood culture isolates. Eur J Clin Microbiol Infect Dis. 2000;19: 39–42. 10706178 10.1007/s100960050007

[31] Chai WL, Hamimah H, Cheng SC, Sallam AA, Abdullah M. Susceptibility of *Enterococcus faecalis* biofilm to antibiotics and calcium hydroxide. J Oral Sci. 2007;49: 161–166. 17634730 10.2334/josnusd.49.161

[32] Sena NT, Gomes BP, Vianna ME, Berber VB, Zaia AA, Ferraz CC et al In vitro antimicrobial activity of sodium hypochlorite and chlorhexidine against selected single-species biofilms. Int Endod J. 2006;39: 878–85. 17014526 10.1111/j.1365-2591.2006.01161.x

[33] CLSI. Methods for Dilution Antimicrobial Susceptibility Tests f or Bacteria That Grow Aerobically; Approved Standard, Ninth Edition CLSI document M07-A9. Wayne, PA: Clinical and Laboratory Standards Institute; 2012.

[34] Sedgley C, Buck G, Appelbe O. Prevalence of *Enterococcus faecalis* at multiple oral sites in endodontic patients using culture and PCR. J Endod. 2006;32: 104–109. 16427455 10.1016/j.joen.2005.10.022

[35] Sedgley C, Nagel AC, Shelburne DB, Clewell DB, Appelbe O, Molander A. Quantitative real-time PCR detection of oral *Enterococcus faecalis* in humans. Arch of Oral Biol. 2005;50: 575–83. 15848151 10.1016/j.archoralbio.2004.10.017

[36] Zoletti GO, Siqueira JF Jr, Santos KRN. Identification of *Enterococcus faecalis* in root-filled teeth with or without periradicular lesions by culture-dependen and independent approaches. J Endod. 2006;32, 722–8. 16861069 10.1016/j.joen.2006.02.001

[37] Foschi F, Cavrini F, Montebugnoli L, Stashenko P, Sambri V, Prati C. Detection of bacteria in endodontic samples by polymerase chain reaction assays and association with defined clinical signs in Italian patients. Oral Microbiol Immunol. 2005;20: 289–95. 16101964 10.1111/j.1399-302X.2005.00227.x

[38] Franzetti L, Pompei M, Scarpellini M, Galli A. Phenotypic and genotypic characterization of *Enterococcus* spp. of different origins. Curr Microbiol. 2004;49: 255–60. 15386113 10.1007/s00284-004-4242-6

[39] Mihaila-Amrouche L, Schlegel L, Bouvet A, Association for l’Etude de la prevention de l’endocardite infectieuse (AEPEI) study group. Impact of susceptibility to antibiotics streptococci & enterococci isolated from patients with infective endocarditis on antibiotic treatment. Indian J Med Res. 2004;199: 80–83. 15232168

[40] Garcia-Garrote F, Cercenado E, Bouza E. Evaluation of a new system, VITEK 2, for identification and antimicrobial susceptibility testing of enterococci. J Clin Microbiol. 2000;38: 2108–11. 10834961 10.1128/jcm.38.6.2108-2111.2000 PMC86739

[41] Winston LG, Pang S, Haller BL, Wong M, Chambers HF III, Perdreau-Remington F. API 20 Strep identification system may incorrectely speciate enterococci with low level resistance to vancomycin. Diagn Microbiol Infect Dis. 2004;48, 287–288. 15062923 10.1016/j.diagmicrobio.2003.10.008

[42] André P, Metzger C, Petey S, Muller D, Vidon DJM. Chemiluminescence of enterococci isolates from freshwater. FEMS Microbiol Lett 2005;245:123–129. 15796989 10.1016/j.femsle.2005.02.036

[43] Miranda JM, Franco CM, Vásquez BI, Fente CA, Barros-Velázquez J, Cepeda A. Evaluation of Chromocult^®^ enterococci agar for the isolation and selective enumeration of *Enterococcus* spp. in broilers. Lett Applied Microbiol. 2005;41: 153–6. 10.1111/j.1472-765X.2005.01728.x 16033513

[44] Araya M, Davidovich G, Chaves C, Arias ML. Identificación de *Enterococcus* sp. en muestras de leche cruda del Área Metropolitana de Costa Rica y evaluación del patrón de sensibilidad a antibióticos. Arch Latinoam Nutricion. 2005;2: 161–166. 16335226

[45] Vidana R, Rashid MU, Özenci V, Weintraub A, Lund B. The origin of endodontic *Enterococcus faecalis* explored by comparison of virulence factor patterns and antibiotic resistance to that of isolates from stool samples, blood cultures and food. Int Endod J. 2016;49(4):343–51. 10.1111/iej.12464 25950381

[46] Roberts AP, Mullany P. Oral biofilms: a reservoir of transferable, bacterial, antimicrobial resistance. Expert Rev Anti Infect Ther. 2010;8: 1441–1450. 10.1586/eri.10.106 21133668

